# Prenatal PCB-153 Exposure and Decreased Birth Weight: Verner et al. Respond

**DOI:** 10.1289/ehp.1307796R

**Published:** 2014-04-01

**Authors:** Marc-André Verner, Melvin E. Andersen, Harvey J. Clewell, Matthew P. Longnecker

**Affiliations:** 1 Department of Environmental Health, Harvard School of Public Health, Boston, Massachusetts, USA; 2 Institute of Environmental Medicine, Karolinska Institutet, Stockholm, Sweden; 3 The Hamner Institutes for Health Sciences, Research Triangle Park, North Carolina, USA; 4 Epidemiology Branch, National Institute of Environmental Health Sciences, National Institutes of Health, Department of Health and Human Services, Research Triangle Park, North Carolina, USA

In our recent paper (Verner et al. 2013), we suggested that gestational weight gain (GWG) confounded the association between prenatal PCB-153 (polychlorinated biphenyl-153) exposure and birth weight observed in the meta-analysis by Govarts et al. (2012). Govarts et al. have now performed additional analyses in which they accounted for GWG in cohorts where this information could be abstracted from medical records or questionnaires. Their effort is commendable and sheds light on results from both our paper and their meta-analysis. In line with our findings, they reported that adjusting for GWG in statistical models of PCB-153 and birth weight substantially reduced the strength of the association, without completely attenuating it.

In Govarts et al.’s reanalysis, adjustment for GWG resulted in a reduction in the association from a 293-g decrease [95% confidence interval (CI): –536, –50] in birth weight per 1-µg/L increase in cord serum PCB-153 to 153 g (95% CI: –340, 30), which represents a 48% reduction in the effect estimate. In comparison, our analyses using pharmacokinetic modeling suggested that 79% of the 150-g (95% CI: –240, –50) reduction in birth weight per 1-µg/L increase in cord serum PCB-153 reported in the original paper by Govarts et al. (2012) may be attributable to GWG. It is difficult to speculate how much more attenuation of the PCB-153–birth weight association would occur if it were possible to accurately measure gain in fat mass during pregnancy—the underlying, true confounder—and adjust for it. The data presented by Butte et al. (2003) show that only 57.8% of the variability in the amount of fat mass gained in pregnancy is explained by GWG. Sensitivity analyses to assess the influence of such an improved adjustment would be of interest.

In their letter, Govarts et al. touched on the assumptions of pharmacokinetic models. Both meta-analyses of observational data (Greenland and O’Rourke 2008) and pharmacokinetic simulations of epidemiologic associations have their strengths, and both can produce results that may appear more precise and conclusive than warranted. In those settings where both approaches can be applied, their results can be combined and used to make an improved inference about an association that neither method can definitively quantify. The example of PCB-153 and birth weight in which confounding by GWG was revealed by pharmacokinetic modeling and subsequently accounted for in statistical models demonstrates how these two complementary approaches can work hand in hand to generate more robust effect estimates.

Whether some causal (or perhaps any) association exists between environmental PCB-153 exposure and birth weight is probably beyond the resolution of currently available or practicable methods.
